# Analysis of HFMD Transmissibility Among the Whole Population and Age Groups in a Large City of China

**DOI:** 10.3389/fpubh.2022.850369

**Published:** 2022-04-11

**Authors:** Peihua Li, Jia Rui, Yan Niu, Fang Xie, Yifang Wang, Zhuoyang Li, Chan Liu, Shanshan Yu, Jiefeng Huang, Li Luo, Bin Deng, Weikang Liu, Tianlong Yang, Qun Li, Tianmu Chen

**Affiliations:** ^1^State Key Laboratory of Molecular Vaccinology and Molecular Diagnostics, School of Public Health, Xiamen University, Xiamen, China; ^2^Chinese Center for Disease Control and Prevention, Public Health Emergency Center, Beijing, China

**Keywords:** HFMD, children, mathematical model, age group, infectious diseases

## Abstract

**Background:**

Hand-Foot-and-Mouth-Disease (HFMD) has been widely spread in Asia, and has result in a high disease burden for children in many countries. However, the dissemination characteristics intergroup and between different age groups are still not clear. In this study, we aim to analyze the differences in the transmissibility of HFMD, in the whole population and among age groups in Shenzhen city, by utilizing mathematical models.

**Methods:**

A database that reports HFMD cases in Shenzhen city from January 2010 to December 2017 was collected. In the first stage, a Susceptive-Infected-Recovered (*SIR*) model was built to fit data of Shenzhen city and its districts, and *R*_*eff*_ was used to assess transmissibility in each district. In the second stage, a cross-age groups SIR model was constructed to calculate the difference in transmissibility of reported cases among three age groups of EV71 virus: 0–3 years, 3–5 years, and over 5 years which was denoted as age group 1, 2, and 3, respectively.

**Results:**

From 2010 to 2017, 345,807 cases of HFMD were reported in Shenzhen city, with peak incidence in spring and autumn in Shenzhen city and most of its districts each year. Analysis of the EV71 incidence data by age group revealed that age Group 1 have the highest incidence (3.13 ×10^−7^–2.31 ×10^−4^) while age group 3 had the lowest incidence (0–3.54 ×10^−5^). The differences in weekly incidence of EV71 between age groups were statistically significant (*t*_12_ = 7.563, *P* < 0.0001; *t*_23_ = 12.420, *P* < 0.0001; *t*_13_ = 16.996, *P* < 0.0001). The *R*^2^ of the *SIR* model Shenzhen city population-wide HFMD fit for each region was >0.5, and *P* < 0.001. *R*_*eff*_ values were >1 for the vast majority of time and regions, indicating that the HFMD virus has the ability to spread in Shenzhen city over the long-term. Differences in *R*_*eff*_ values between regions were judged by using analysis of variance (ANOVA) (*F* = 0.541, *P* = 0.744). *S*_*i*_*I*_*i*_*R*_*i*_*-S*_*j*_*I*_*j*_*R*_*j*_ models between age groups had *R*^2^ over 0.7 for all age groups and P <0.001. The *R*_*eff*_ values between groups show that the 0–2 years old group had the strongest transmissibility (median: 2.881, range: 0.017–9.897), followed by the over 5 years old group (median: 1.758, range: 1.005–5.279), while the 3–5 years old group (median: 1.300, range: 0.005–1.005) had the weakest transmissibility of the three groups. Intra-group transmissibility was strongest in the 0–2 years age group (median: 1.787, range: 0–9.146), followed by Group 1 to Group 2 (median: 0.287, range: 0–1.988) and finally Group 1 to Group 3 (median: 0.287, range: 0–1.988).

**Conclusion:**

The incidence rate of HFMD is high in Shenzhen city. In the data on the incidence of EV71 in each age group, the highest incidence was in the 0–2 years age group, and the lowest incidence was in the over 5 years age group. The differences in weekly incidence rate of EV71 among age groups were statistically significant. Children with the age of 0–2 years had the highest transmissibility.

## Background

Hand-Foot-and-Mouth Disease (HFMD) has been a health concern in Asia since the late 1990s (1), with outbreaks reported in Malaysia, Japan, Singapore, Vietnam, and Cambodia (2–4). HFMD has a high disease burden in Asia, with 358,764 cases of HFMD in Japan each year (5). Also, it remains a major health problem for children in China, affecting more than 2 million children each year. Especially, Guangdong Province has been one of the most severely affected provinces (6). The annual incidence of HFMD in Guangdong Province exceeds 30 cases in every 10,000 people and case number has accounted for approximately 15% of the total number of cases in China in recent years (7). In addition, Shenzhen city had the highest number of HFMD cases among 143 cities in mainland China between 2009 and 2014 (8).

HFMD is a viral disease that has received great attention in the last two decades because of its high incidence rate in the pediatric population. The clinical presentation of HFMD is characterized by fever and a blistering rash, mostly on the hands, feet and oral mucosa (9). The disease is generally mild and self-limiting, but neurologic and cardiopulmonary-related complications may occur in disease outbreaks (10). Among all common febrile and rash illnesses (11), HFMD has remained the one that most frequently affects young children (12, 13). A series of analyses have shown that children younger than 5 years old are more likely to develop HFMD and that it is more likely to accumulate in younger children (14–16).

The main pathogens of HFMD infection are enterovirus 71 (EV71) and coxsackievirus A16 (CVA16) (14, 17), and EV71 infection is particularly the main viral subtype causing severe and fatal cases (12).

However, the mechanism by which EV71 causes severe central nervous system complications is still unclear. It is suggested that this may be due to a combination of pathologic immune response and direct viral action, but there is no effective treatment and no biomarker that can be used as an early warning of severe HFMD. In terms of vaccines, Asian countries that have experienced a history of HFMD infection and pandemics are actively developing vaccines as a preventive measure. Among them, a vaccine for EV-A71 in China has been available in 2016 (9, 10), and a bivalent EV-A71 / CV-A16 vaccine should enter clinical trials in the near future (13). The research of Joseph T. Wu et al. showed that the cost of routine EV71 vaccination is cost-effective in China (15). However, it is promoted as a class II vaccine in China, causing some limitations in terms of immunization coverage.

Currently, many mathematical models have also been applied to the study of HFMD, such as the application of Bayesian spatio-temporal models to analyze the factors influencing HFMD and the effects of interventions (16, 18, 19), the application of lagged nonlinear models to assess the effects of meteorological factors on incidence (20, 21). SIR models (22–24), SEIR models (25), SEILR models (26), and other models (27, 28) are also used in the study of HMFD. In addition, some scholars have also focused on the differences in HMFD between different age groups (29, 30). It indicates that the existing studies related to HFMD still focus mostly on spatial and temporal variation (11, 31), the expression of overall epidemiological characteristics and the relationship between meteorological factors and the incidence of HFMD, while there is a lack of studies targeting the analysis of EV71 virus by age groups.

In this study, we assumed that there is variability in the transmissibility among the major pathogens of HFMD and variability and possible interaction in the transmission of EV71 virus among different age groups. Based on this hypothesis, we collected data on reported HFMD cases in a city with a high prevalence of HFMD in China (Shenzhen city), grouped the cases by age according to their basic information, and then used a seasonally adjusted SIR model to calculate transmissibility of different pathogens among different age groups, and further investigated the magnitude of all-virus transmissibility of HFMD in Shenzhen city. Besides, in this paper, we studied for the first time the characteristics of the transmission pattern of HFMD among different age groups in the whole population, established a transmission model of EV71 virus transmission among different age groups, and analyzed the reasons for the differences between groups.

## Materials and Methods

### Data Collection

In this study, we collected HFMD cases from January 2010 to December 2017 in Shenzhen city, Guangdong Province, including the number of reported cases and deaths per day, age, sex, exposure history, date of onset, and severity of disease, and obtained basic information on the population of Shenzhen city, including the year-end resident, birth rate and death rate, by searching the Shenzhen city Statistical Yearbook 2010–2017. Between 2010 and 2017, the population exceeded 12 million, and the average birth rate was 18.63 per 1,000 (range: 16.41/1,000–21.46/1,000) and the median death rate was 7.32 per 1,000 (range: 6.19/1,000–9.72/1,000). When we come to the Population Composition of Shenzhen city, the number of people in each age group increased linearly from 2014 to 2017, but the composition ratio remained stable: the 0–2 years old group remained stable around 2.611% (range: 2.609–2.613%), the 3–5 years old group remained stable around 2.163% (range: 2.161–2.165%) and the over 5 years old group remained stable around 95.225% (range: 95.222–95.230%).

The population of the 0–2 years old group was 281.240 thousand, 297.156 thousand, 310.928 thousand and 327.383 thousand from 2014 to 2017 respectively. The population of the 3–5 years old group from 2014 to 2017 was 232.886 thousand, 246.202 thousand, 271.202 thousand and 271.202 thousand respectively. The population of the over 5 years old group from 2014 to 2017 was 1024.789 thousand, 10,835.555 thousand, 11,929.736 thousand and 11,929.736 thousand respectively.

Shenzhen city is a large city located in the south of the coastal area of Guangdong Province, China. Shenzhen city has a southern subtropical monsoon climate with long summers and short winters, mild climate, abundant sunshine and rainfall, and an annual average temperature of 23.0°C. The climate is strongly influenced by the monsoon, with “warm temps” and humid weather in spring, high temperatures and rain in summer, little rain in fall with drought and often typhoons, and short and dry winters with little rain (32). Overall, it is a city with long summers and short winters, strongly influenced by the monsoon climate.

### Model Introduction

#### *SIR* Model

**Figure 1:**
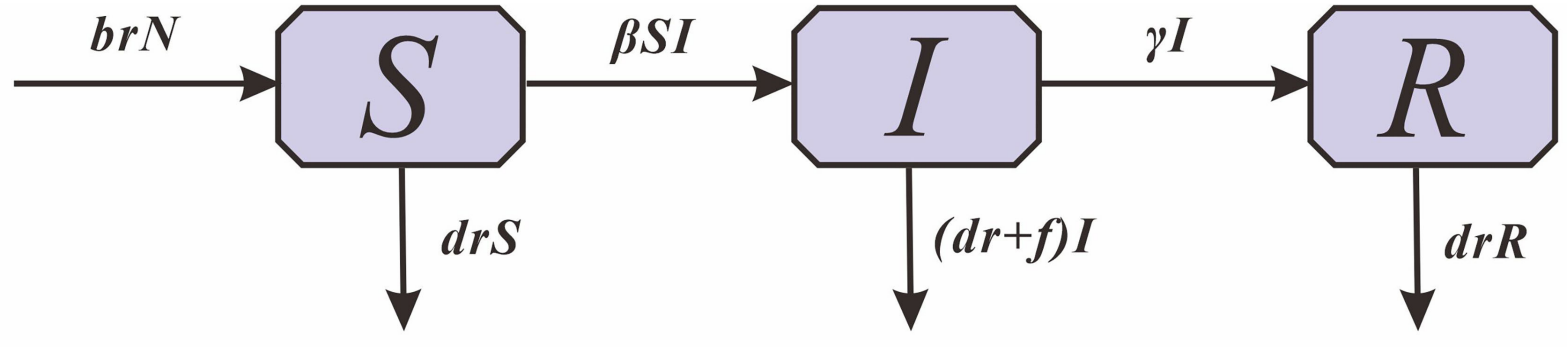
The Susceptive-Infected-Recovered model.

Based on the transmission mechanism of HFMD and the type of case report data, the Susceptible -Infectious -Removed (*SIR*) model was used (Figure 1).

In this model, *S* denotes susceptible individuals, *I* denotes infectious individuals, *R* denotes recovered individuals and *N* denotes the total population size. The parameters *br, dr, f* , β and γ refer to the natural birth rate of the population, the mortality rate of the population, the mortality rate of HFMD, the relative transmission rate and the relative recovery rate, respectively.


$$\begin{array}{l} \frac{dS}{dt}=brN-\beta SI-drS\\ \frac{dI}{dt}=\beta SI-\gamma I-\left(dr+f\right)I\\ \frac{dR}{dt}=\gamma I-drR\\ N=S+I+R \end{array}$$

#### *S*_*i*_*I*_*i*_*R*_*i*_-*S*_*j*_*I*_*j*_*R*_*j*_ Model

**Figure 2:**
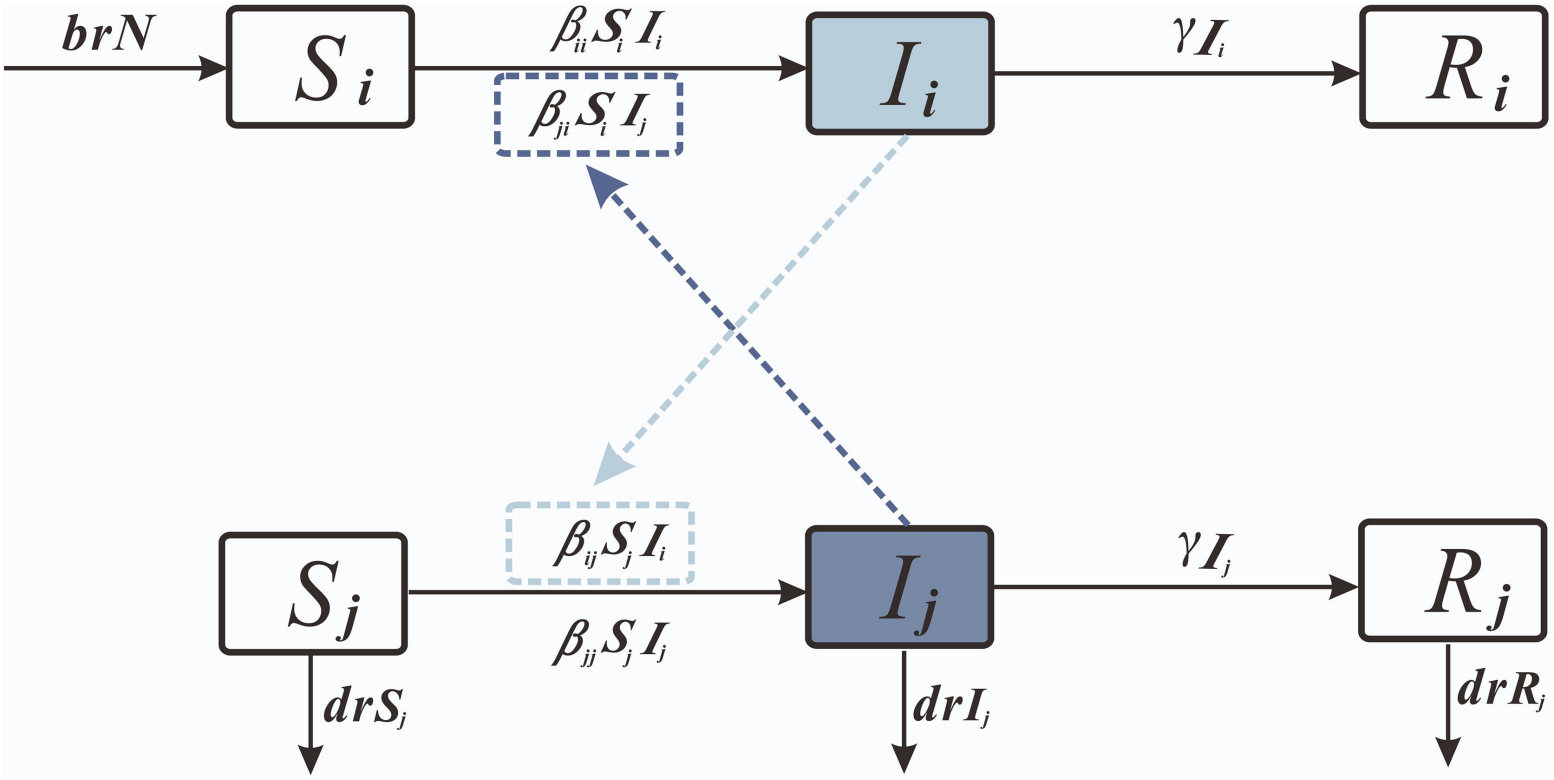
The cross-age groups Susceptive-Infected-Recovered model.

Considering the interactions between age groups, we created the age group *SIR* model (Figure 2).

We set 0–2 years old as age group 1; 3–5 years old as age group 2; and over 5 years old as age group 3.


$$\begin{array}{l} \frac{dS_{1}}{dt}=brN-\beta_{11}S_{1}I_{1}-\beta_{21}S_{1}I_{2}-\beta_{31}S_{1}I_{3}\\ \frac{dI_{1}}{dt}=\beta_{11}S_{1}I_{1}+\beta_{21}S_{1}I_{2}+\beta_{31}S_{1}I_{3}-\gamma^{\prime }I_{1}\\ \frac{dR_{1}}{dt}=\gamma^{\prime }I_{1} \end{array}$$

$$\begin{array}{l} \frac{dS_{2}}{dt}=-\beta_{22}S_{2}I_{2}-\beta_{12}S_{2}I_{1}-\beta_{32}S_{2}I_{3}\\ \frac{dI_{2}}{dt}=\beta_{22}S_{2}I_{2}+\beta_{12}S_{2}I_{1}+\beta_{32}S_{2}I_{3}-\gamma^{\prime }I_{2}\\ \frac{dR_{2}}{dt}=\gamma^{\prime }I_{2} \end{array}$$

$$\begin{array}{l} \frac{dS_{3}}{dt}=-\beta_{33}S_{3}I_{3}-\beta_{13}S_{3}I_{1}-\beta_{23}S_{3}I_{2}-drS_{3}\\ \frac{dI_{3}}{dt}=\beta_{33}S_{3}I_{3}+\beta_{13}S_{3}I_{1}+\beta_{23}S_{3}I_{2}-\gamma^{\prime }I_{3}-drI_{3}\\ \frac{dR_{3}}{dt}=\gamma^{\prime }I_{3}-drR_{3} \end{array}$$

$$\begin{array}{l} N=S_{1}+S_{2}+S_{3}+I_{1}+I_{2}+I_{3}+R_{1}+R_{2}{+R}_{3} \end{array}$$

### Parameter Estimation

As shown in Table 1, there are eight parameters (β_1_, γ, *br, dr*, and *f* ).

1) The propagation coefficient among individuals in the *SIR* model is set to β. β = 0.340609 is obtained by fitting the reported data of Shenzhen city.
2) The parameters *br, dr*, and *f* (33) were calculated based on finding the Shenzhen city Yearbook and the collected data. The annual *br* and *dr* values were collected; the weekly values of the two parameters were calculated; and the weekly *br* and *dr* values were 0.000352 (range: 0.000330–0.000383) and 0.0000129 (range: 0.0000127–0.0000187). The references give the susceptibility of the population and the likelihood of the population and the infectiousness of the population were 0.67, 0.4, and 0.433, respectively.
3) The age group *SIR* model in which *f*_1_, *f*_2_, and f_3_ denote the disease and death rates in the three age groups 0–2 years, 3–5 years, and over 5 years, respectively, were taken as 0.0003. β_11_ denotes the transmission coefficient among individuals in age group 1; β_22_ denotes the transmission coefficient among individuals in age group 2 minus; β_12_ denotes the transmission coefficient from individuals in age group 1 to individuals in age group 2; β_13_ denotes the transmission coefficient from individuals in age group 1 to individuals in age group 2; and β_14_ denotes the transmission coefficient from individuals in age group 1 to individuals in age group 2. Propagation coefficient from individuals within age group 1 to individuals in age group 3; β_21_ denotes the propagation coefficient from individuals within age group 2 to individuals in age group 1; β_23_ denotes the propagation coefficient from individuals within age group 2 to individuals in age group 3; β_31_ denotes the propagation coefficient from individuals within age group 3 to individuals in age group 1; and β_32_ denotes the propagation coefficient from individuals within age group 3 to individuals in age group 2. The *R*_*eff*_ subscript corresponds to the same age group object.
4) According to the seasonal cyclic pattern of the incidence data, they were divided into 18 segments, and β were fitted separately within and between each age group.

**Table 1:** Parameter estimation.

**Parameter**	**Description**	**Value**	**Range**	**Method**
β	Inter-individual propagation coefficient	Seen text	≥0	Curve fitting
γ	Relative recovery rate	1/14	-	References
*br*	Population birth rate	3.52 × 10^−4^	3.30 × 10^−4^-3.83 × 10^−4^	Analysis on the reported data
*dr*	Population mortality rate	1.29 × 10^−5^	1.27 × 10^−5^-1.87 × 10^−5^	Analysis on the reported data
*f*	Case fatality rate	0.0003	0–1	References

### Indicators of Transmission Capacity

The basic reproduction number is an important parameter to determine whether the disease is epidemic or not, which refers to the number of new cases expected to be directly transmitted by one infectious agent in the susceptible population during its transmission period. Effective reproduction number (*R*_*eff*_) was set as the evaluation index, i.e., the basic reproduction number after intervention, to evaluate the impact of the intervention on the relative transmission capacity of HFMD among the population. The calculation process of the *R*_*eff*_ equation can be seen whereupon:

Step 1: Divide the derivatives of all infected compartments into two parts: the first part represents F new infections and the other part V represents the transformation between non-new compartments.


$$\begin{array}{l} \frac{d}{dt}\left(\begin{array}{c} I_{1}\\ I_{2}\\ I_{3} \end{array}\right)=\left(\begin{array}{c} S_{1}=\left(I_{1}\beta_{11}+I_{2}\beta_{21}+I_{3}\beta_{31}\right)\\ S_{2}=\left(I_{1}\beta_{12}+I_{2}\beta_{22}+I_{3}\beta_{32}\right)\\ S_{3}=\left(I_{1}\beta_{13}+I_{2}\beta_{23}+I_{3}\beta_{33}\right) \end{array}\right)-\left(\begin{array}{c} I_{1}\gamma \\ I_{2}dr+I_{2}\gamma \\ I_{3}dr+I_{3}\gamma \end{array}\right)\\ \;\overset{def}{=}F-V \end{array}$$

Step 2: Taking the derivatives of the vectors F and V with respect to the variable [*I*_1_ *I*_2_ *I*_3_], respectively, yields the corresponding Jacobi matrices F and V :


$$\begin{array}{l} F=\left(\begin{array}{ccc} S_{1}\beta_{11} & S_{1}\beta_{21} & S_{1}\beta_{31}\\ S_{2}\beta_{12} & S_{2}\beta_{22} & S_{2}\beta_{32}\\ S_{3}\beta_{13} & S_{3}\beta_{23} & S_{3}\beta_{33}\\ ‏ \end{array}\right)\\ V=\left(\begin{array}{ccc} \gamma & 0 & 0\\ 0 & dr+\gamma & 0\\ 0 & 0 & dr+\gamma \\ ‏ \end{array}\right) \end{array}$$

and further calculate the V ^−1^:


$$\begin{array}{l} V^{-1}=\left(\begin{array}{ccc} \frac{1}{\gamma } & 0 & 0\\ 0 & \frac{1}{dr+\gamma } & 0\\ 0 & 0 & \frac{1}{dr+\gamma }\\ ‏ \end{array}\right) \end{array}$$

Step 3: Calculate the next generation matrix *M* = F V ^−1^. The elements in row j and column i of the matrix *M* are the *R*_*eff, ij*._ of the intergroup interactions. The result of the calculation of *R*_*ij*_ can be obtained by replacing *S*_*i*_=*N*_*i*_ into the equation.


$$\begin{array}{l} M=\left(\begin{array}{ccc} \frac{S_{1}\beta_{11}}{\gamma } & \frac{S_{1}\beta_{21}}{dr+\gamma } & \frac{S_{1}\beta_{31}}{dr+\gamma }\\ \frac{S_{2}\beta_{12}}{\gamma } & \frac{S_{2}\beta_{22}}{dr+\gamma } & \frac{S_{2}\beta_{32}}{dr+\gamma }\\ \frac{S_{3}\beta_{13}}{\gamma } & \frac{S_{3}\beta_{23}}{dr+\gamma } & \frac{S_{3}\beta_{33}}{dr+\gamma }\\ ‏ \end{array}\right) \end{array}$$

The elements in row j and column *i* of the matrix *M* are the *R*_*eff, ij*._ of the intergroup interactions. The result of the calculation of *R*_*ij*_ can be obtained by replacing *S*_*i*_=*N*_*i*_ into the equation.

Step 4: *R*_0_ is defined as the maximum eigenvalue of *M*, whereupon:


$$\begin{array}{l} R_{0}=\lambda_{\max}\left(M\right) \end{array}$$

The analytic expressions for the eigenvalues of the matrix *M* are particularly complex. The following approach is taken for this purpose.

1) First calculate the value of *R*_*ij*_ and then the maximum eigenvalue of the numerical matrix *M*.
2) Use the definition method to calculate the overall *R*_0_.
$$\begin{array}{l} R_{0}=\overset{n}{\underset{i=1}{\sum }}\left[P\left(x_{0}\in N_{i}\right)\overset{n}{\underset{i=1}{\sum }}R_{ij}\right] \end{array}$$

### Software Introduction

Berkeley Madonna 8.3.18 (developed by Robert Macey and George Oster of the University of California at Berkeley. Copyright ©1993–2001 Robert I. Macey & George F. Oster) ran model coefficients estimated by curve fitting to the data and simulated the effects of the intervention. SPSS 13.0 (IBM Corp., Armonk, NY, USA) was used for statistical testing, with *t*-tests used to calculate differences between age groups and *R*^2^ used to assess curve fitting.

## Results

### Epidemiological Features

From 2010 to 2017, 345,807 cases of HFMD were reported in Shenzhen city, including 129,812 cases in Baoan District, 26,377 cases in Luohu District, 24,008 cases in Futian District, 23,009 cases in Nanshan District, 136,832 cases in Longgang District, and 5,769 cases in Yantian District. Among them, 233,895 cases were 0–2 years old, 98,911 cases were 3–5 years old, and 16,071 cases were over 5 years old. Among them, 16 cases died, and the mortality rate was 0.0059%.

**Figure 3:**
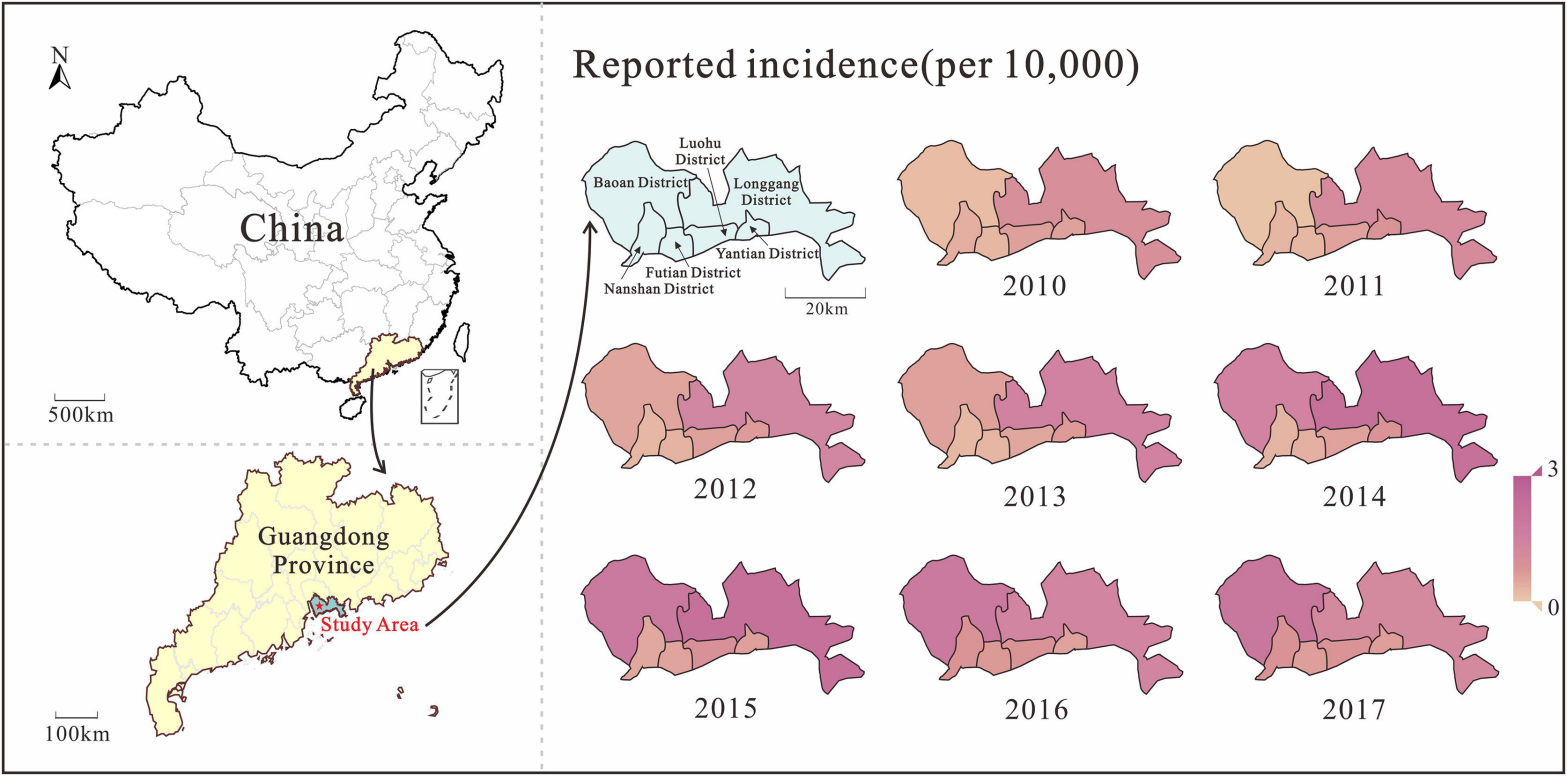
The Map of HMFD burden distribution in Shenzhen city.

Analyzing the incidence data of Shenzhen city from 2010 to 2017, the median incidence rate was 5.26/100,000 people. The lowest incidence rate was 0.11/100,000 people in week 4 of 2011; the highest incidence rate was 45.10/100,000 people in week 39 of 2017. From the spatio-temporal distribution map (Figure 3), it can be seen that the burden of HMFD in Shenzhen city gradually increased from 2010 to 2017, and the increase in incidence rate was mainly concentrated in Baoan and Longgang districts, among which Longgang district had a more obvious trend of rising first and then decreasing. In addition, after 2013, the burden of disease in Nanshan District, Futian District, Luohu District, and Yantian District differed significantly from Baoan District and Longgang District, and there were also slight fluctuations during the period.

**Figure 4:**
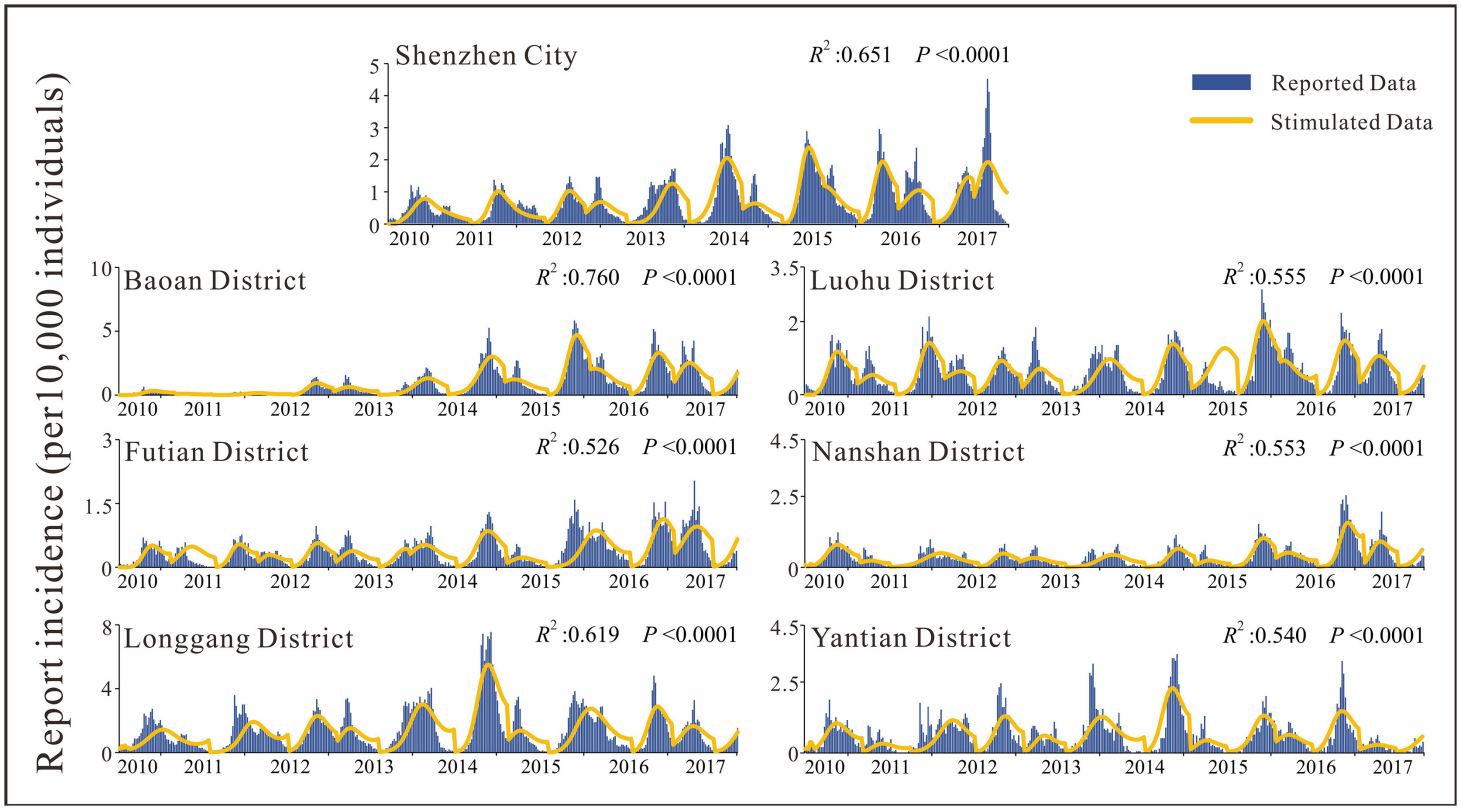
Fitting results for HMFD incidence rates in Shenzhen city and its districts.

By analyzing the weekly reported case data, there are approximately two epidemic cycles per year, alternating seasonally from spring to summer and from summer to autumn. Except for 2013, Shenzhen city and most of its districts had peak incidence in both spring and autumn, especially in Luohu and Yantian districts, where the peak incidence in spring tended to be higher than that in autumn (Figure 4). Among the districts, Baoan, Longgang and Yantian had higher incidence rates, with Longgang having the highest incidence rate (1.28 × 10^−2^-7.53 × 10^−4^) and Futian having the lowest incidence rate (0–2.75 × 10^−4^). In 2017, Baoan district had the highest incidence rate in Shenzhen city, with an incidence rate of 87.36/100,000 people. The differences in incidence rates among districts were statistically significant as calculated by ANOVA (*F* = 65.006, *P* < 0.0001). And the results of multiple comparisons in different districts were showed in Table 2.

**Table 2:** The *P* values of multiple comparisons for different districts (LSD).

**Comparison groups**	**Baoan district**	**Futian district**	**Nanshan district**	**Yantian district**	**Luohu district**	**Longgang district**
Baoan district						
Futian district	0					
Nanshan district	0	0				
Yantian district	0	0.0002	0			
Luohu district	0	0.7209	0	0		
Longgang district	0	0.0006	0	0.7404	0.0020	

*α = 0.05*.

**Figure 5:**
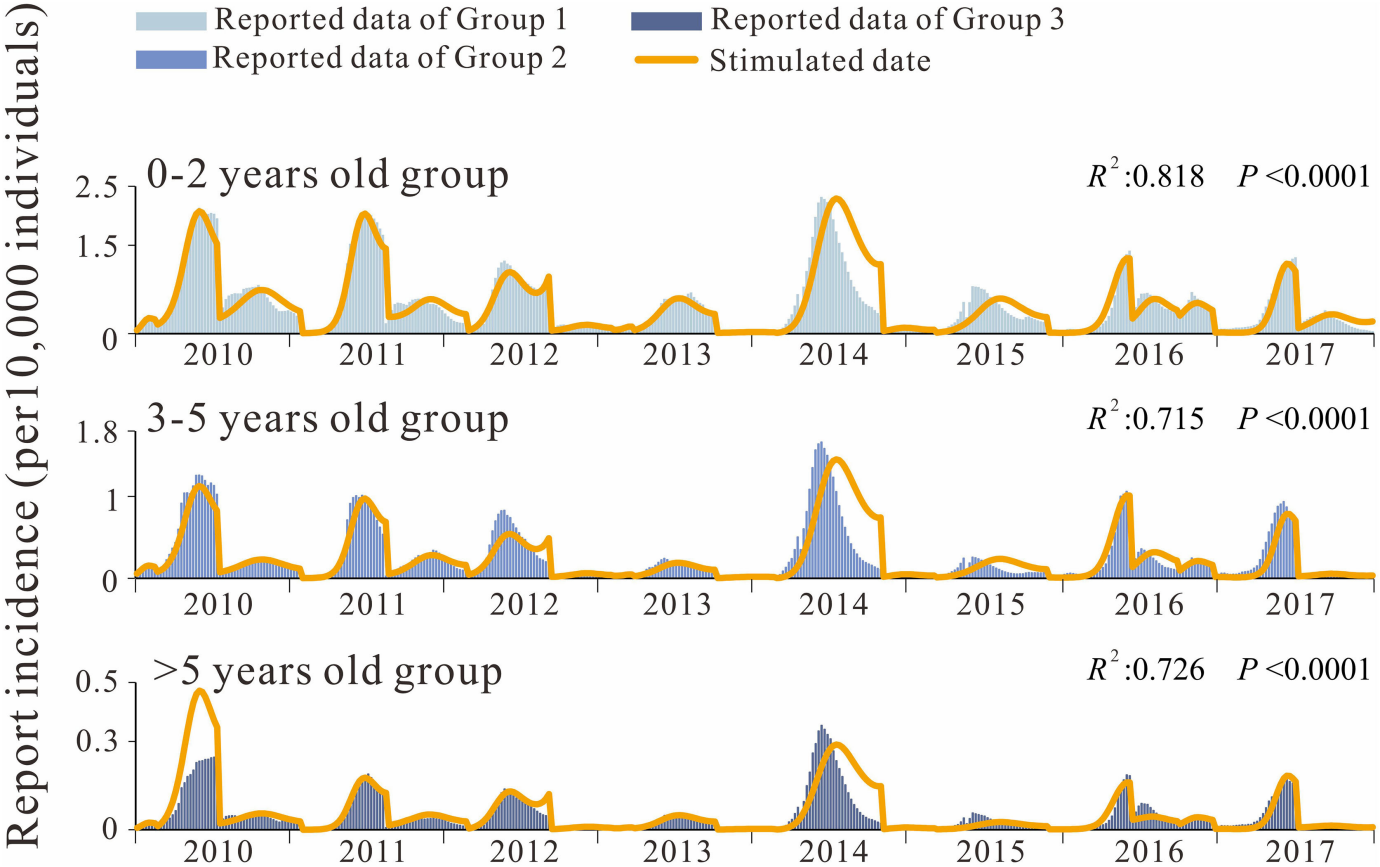
Fitting results for EV71 incidence rates by age group.

Analysis of the incidence data by age group also revealed a peak in spring and autumn, with a significantly higher peak in spring than in autumn. Incidence rates were the highest (3.13 × 10–7 to 2.31 × 10–4) in group 1 (the 0–2 years age group) and the lowest (0–3.54 × 10–5) in group 3 (the over 5 years age group), with the incidence in group 1 being 10.02 times higher than that in the group 3 (Figure 5). The differences in weekly EV71 prevalence between age groups were statistically significant (*t*_12_ = 7.563, *P* < 0.0001; *t*_23_ =12.420, *P* < 0.0001; *t*_13_ =16.996, *P* < 0.0001;). The composition ratio of the three age groups did not change significantly during 2014–2017, and the median composition ratio of group 2 was 65.267% (59.654–74.922%), the median composition ratio of group 2 (the 3–5 years group) was 29.875% (20.385–33.895%), and the median composition ratio of group 3 was 5.197% (4.155–6.452%), all fluctuating within a certain range.

### Model Fitting Effect

#### *SIR* Model

First, we fitted the SIR model for the weekly incidence of population-wide HFMD in Shenzhen city and each sub-district. In the fitting process, segments were fitted to the data. According to the seasonal characteristics of HFMD, each peak period was divided into a segment, with an average of 2–3 segments per year in Shenzhen city. The results of fitting for population-level HFMD in each district of Shenzhen city were good, with *R*^2^ >0.5 and *p* < 0.001 for all districts (Figure 4). The quartiles of reported incidence in Shenzhen city were *P*_25_ = 1.78 × 10^−5^, *P*_50_ = 5.26 × 10^−5^, *P*_75_ = 1.19 × 10^−4^. The total incidence reported in Shenzhen city had an increasing trend year by year, with the highest peak in 2017 (4.51 × 10^−4^), while the remaining districts showed a trend of increasing and then decreasing trend, except for Futian and Nanshan districts.

#### *S*_*i*_*I*_*i*_*R*_*i*_-*S*_*j*_*I*_*j*_*R*_*j*_ Model

We also developed the *S*_*i*_*I*_*i*_*R*_*i*_*-S*_*j*_*I*_*j*_*R*_*j*_ model to fit the HFMD incidence data between age groups in Shenzhen city for 0–2 years, 3-5 years, and over 5 years of age for transmissibility. As can be seen from the fitted curves (Figure 5), the fit was good among the age groups. The fitted *R*^2^ values for each group are listed in the listed fit effect table, and it can be seen that *R*^2^ is over 0.7 for all age groups, and *p* < 0.001 for all (Table 3). The quartiles for age group 1 were *P*_25_ = 1.08 × 10^−5^, *P*_50_ = 3.17 × 10^−5^, *P*_75_ = 6.58 × 10^−5^, age group 2 were *P*_25_ = 4.98 × 10^−6^, *P*_50_ = 1.23 × 10^−5^, *P*_75_ = 2.62 × 10^−5^, and age group 3 were *P*_25_ = 8.26 × 10^−7^, *P*_50_ = 2.26 × 10^−6^, *P*_75_ = 4.97 × 10^−6^. The transmission coefficients β for each age group at each time period were obtained by fitting, and the fitting results are shown in Table 4.

**Table 3:** The *P* values and fitted *R*^2^ values for each group.

**Age groups**	***R* ^2^**	***P***
0–3 years old group	0.818	<0.0001
3–5 years old group	0.715	<0.0001
>5 years old group	0.726	<0.0001

**Table 4:** The weekly transmission coefficients β for each age group.

**Year**	**Weeks**	**β_11_**	**β_22_**	**β_33_**	***β_12_***	***β_13_***	***β_21_***	***β_23_***	***β_31_***	***β_32_***
2010	1–8	0.228	0.245	0.020	0.000	0.000	0.015	0.097	0.030	0.003
	8–29	0.127	0.120	0.004	0.014	0.007	0.069	0.014	0.149	0.002
	29–52	0.128	0.149	0.023	0.001	0.000	0.038	0.023	0.016	0.001
2011	1–5									
	5–33	0.018	0.096	0.009	0.015	0.009	0.190	0.000	0.045	0.005
	34–52	0.260	0.221	0.129	0.009	0.023	0.157	0.011	0.052	0.436
2012	1–8									
	9–36	0.118	0.161	0.018	0.000	0.000	0.044	0.025	0.197	0.000
	37–52	0.079	0.069	0.046	0.001	0.002	0.021	0.001	0.004	0.001
2013	1–5									
	6–12	0.226	0.194	0.024	0.021	0.022	0.182	0.001	0.091	0.075
	13–40	0.076	0.035	0.214	0.108	0.000	0.138	0.000	0.046	0.085
	41–52	0.427	0.294	0.225	0.142	0.031	0.251	0.027	0.083	0.456
2014	1–8									
	9–45	0.254	0.050	0.039	0.114	0.018	0.000	0.003	0.046	0.003
	45–52	0.222	0.145	0.004	0.020	0.010	0.114	0.004	0.123	0.681
2015	1–10									
	11–48	0.001	0.001	0.000	0.000	0.000	0.000	0.000	0.000	0.001
	48–53	0.127	0.008	0.007	0.099	0.006	0.248	0.015	0.006	0.456
2016	1–23									
	24–52	0.242	0.017	0.008	0.099	0.018	0.000	0.012	0.000	0.110
2017	1–27	0.043	0.055	0.001	0.050	0.020	0.151	0.005	0.006	0.076
	27–53	0.000	0.018	0.001	0.026	0.004	1.036	0.016	0.002	0.007

### Assessment of Transmissibility

**Figure 6:**
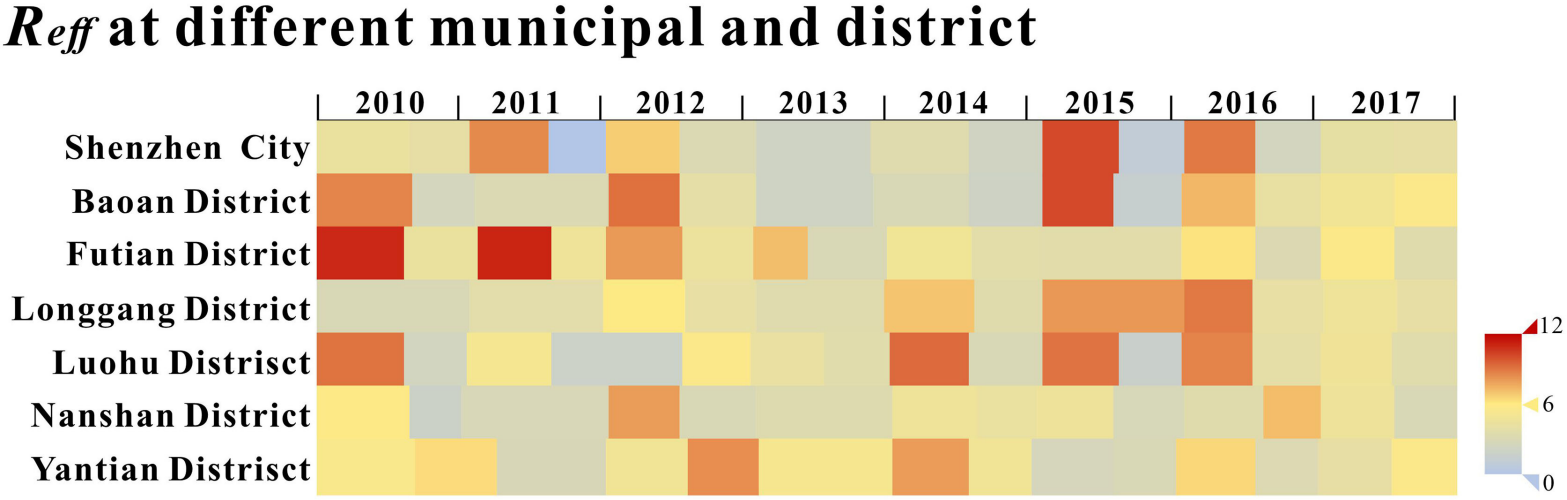
The transimissibility of the first stage.

The calculated *R*_*eff*_ values of Shenzhen city and each district are shown in Figure 6, and it can be seen that the *R*_*eff*_ values for most of the time are >1, indicating that the HFMD virus were likely to continue spreading in the long term.

The median *R*_*eff*_ of Shenzhen city was 4.001 (Interquartile range [IQR]: 0.021–8.929), with *R*_*eff*_ values <1 in the second cycle of 2011; the median *R*_*eff*_ for Baoan was 4.069 (IQR: 2.096–10.139), with *R*_*eff*_ values all over 1; the median *R*_*eff*_ of Longgang District was 4.126 (IQR: 2.935–8.922), with *R*_*eff*_ values all over 1 and a gradual upward trend from 2010 to 2016; the median *R*_*eff*_ of Luohu District had a median *R*_*eff*_ of 4.298 (IQR: 1.730- 9.314), with *R*_*eff*_ values over 1; the median *R*_*eff*_ of Nanshan District was 3.939 (IQR: 1.937- 8.002), with *R*_*eff*_ values all over 1; the median *R*_*eff*_ of Futian District was 4.777 (IQR: 2.924- 11.105), with a decreasing trend of *R*_*eff*_ over 1; the median *R*_*eff*_ in Yantian District was 5.131 (IQR: 1.730- 9.314), with *R*_*eff*_ over 1. *R*_*eff*_ values were slightly <1, indicating that HFMD will continue to be prevalent in Shenzhen city and its districts but will not cause a major outbreak. The differences in *R*_*eff*_ values between districts were not statistically significant (*F* = 0.541, *P* = 0.744) by Analysis of Variance (ANOVA).

The results of the cross-age groups SIR model calculations showed that the 0–2 years age group had the strongest transmissibility (Median[M]: 2.881, IQR: 0.017–9.897), followed by the over 5 years age group (M: 1.758, IQR: 1.005–5.279), while the 3–5 years age group (M:1.300, IQR:0.005–1.005) had the weakest transmissibility among the three groups.

**Figure 7:**
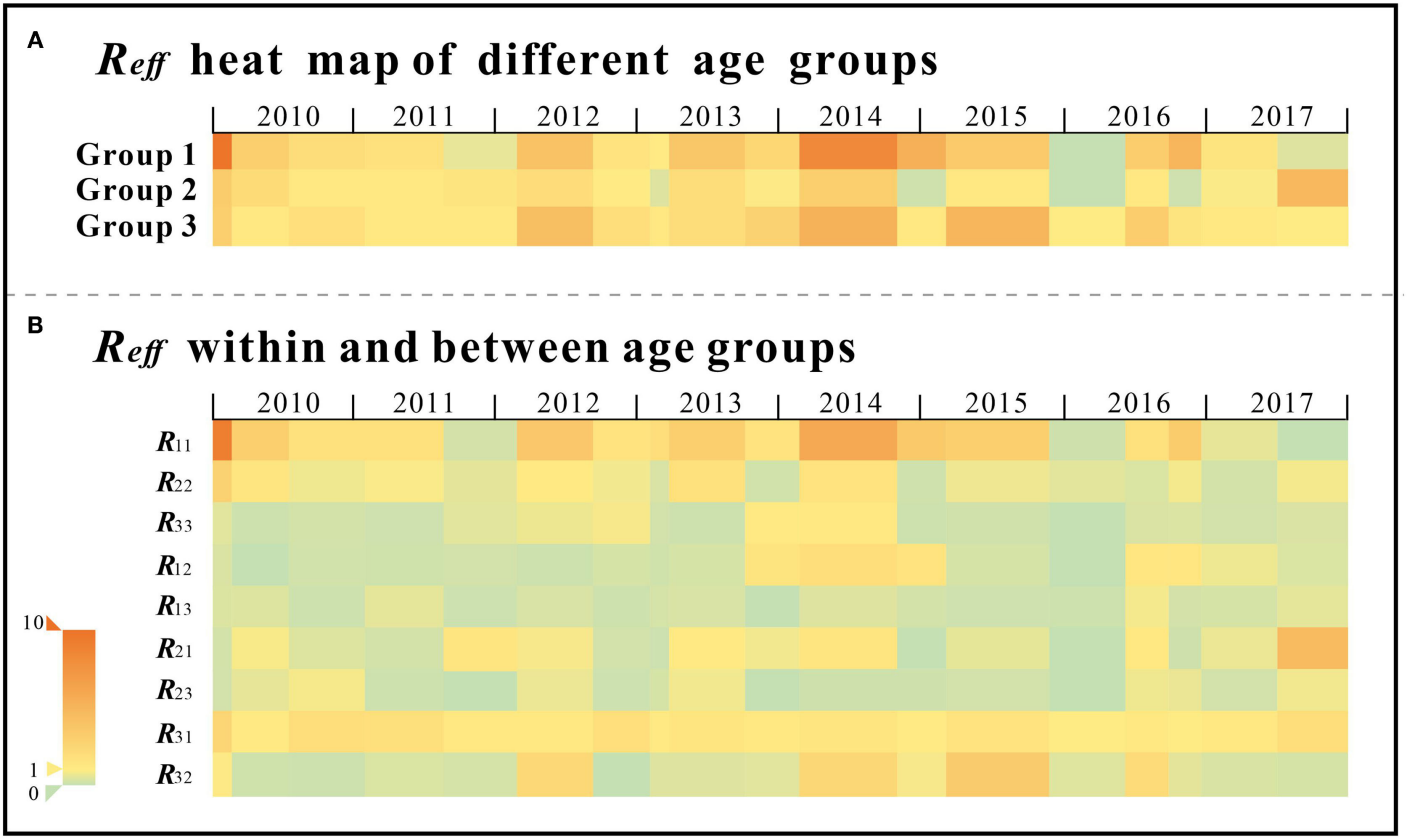
The transimissibility of the second stage **(A)** Group1 indicates the *R*_*eff*_ value of 0–2years old age group; Group 2 indicates the *R*_*eff*_ value of 3–5 years old age group; Group 3 indicates the *R*_*eff*_ value of over 5 years old age group; **(B)** Rij denotes the transmission coefficient from individuals in age group *i* to individuals in age group *j*, which both *i* and *j* take the value 1, 2, 3.

The *R*_*eff*_ for the 0–2 years age group had the largest value in the first half of 2010, followed by a peak in late 2013 and then a gradual decrease; the *R*_*eff*_ for the 3–5 years age group fluctuated within a certain range and had the largest value in the second half of 2017. *R*_*eff*_ values for the over 5 years age showed an increasing and then decreasing trend, peaking at the end of 2013 (Figure 7A).

Intra-group transmissibility was strongest in the 0–2 years age group (*R*_*eff*_: M = 1.787, IQR = 0–9.146), followed by that of Group 1 to Group 2 (*R*__*eff*_12_: M = 0.287, IQR = 0–1.988) and finally Group 1 to Group 3 (*R*_*eff*_: M = 0.287, IQR = 0–1.988 median: 0.287, range: 0–1.988). The *R*_*eff*_ for this intra-group showed a significant decreasing trend during the survey years, and Group 1 to Group 2 showed a trend of increasing before decreasing *R*_*eff*_ and peaked in 2014.

The transmission in the 3–5 years age group was the highest in the intra-group (median: 0.627, range: 0.005–2.928) and Group 2 to Group 1(median: 0.497, range: 0-4.617), and the lowest in the Group 2 to Group 3(median: 0.060, range: 0-0.590); where the intra-group transmissibility of this age group showed a decreasing trend in *R*_*eff*_ year by year.

In the over 5 years age group, the *R*_*eff*_ from Group 3 to Group 1 was the largest and perennially over1 (median: 1.217, range: 1.000–2.619), followed by Group 3 to Group 2(median: 0.063, range: 0–3.412), while intra-group transmission (median: 0.104, range: 0–1.260) was the smallest; where the *R*_*eff*_ from Group 3 to Group 2 showed a trend of rising and then falling and peaked in the second half of 2015, and the intra-group transmission of this age group rose yearly until 2014 peaked and then fell and tended to zero (Figure 7B).

## Discussion

Analysis and observation of the reported incidence of HFMD in Shenzhen city revealed that the incidence rate of HFMD in Shenzhen city is higher than the national-level incidence rate (34, 35), which may be due to the fact that Shenzhen city is a coastal city with a hot and humid climate and a high population density, conditions that favor the reproduction and spread of the virus. In addition, the incidence of HFMD in Asian subtropical countries is characterized by distinct peaks in spring and autumn (36), and the incidence data reported in Shenzhen city are consistent with the double peak at this latitudinal. In this study, we found that the number of reported cases in Shenzhen city increased over time, and the increased incidence suggests that the HFMD pathogen is still spreading in the city. In addition, we found that the four districts with the lowest incidence rates (Nanshan District, Futian District, Luohu District, and Yantian District) also had the top four GDP per capita in Shenzhen city, while the two districts with higher incidence rates had relatively lower GDP per capita in the city's ranking (33). The economic level affects the local sanitation and, therefore, HFMD, as it is an intestinal infectious disease.

There are some limitations in this paper. In terms of model selection, in the past, we also chose the SEIAR model for simulation when cases were reported in a daily reported data (23, 37). However, it is rather unfortunate that exposed and asymptomatic populations were not introduced into the model because of the inability to obtain more precise units of incidence data due to the reporting of HFMD month-based data in Shenzhen. Meanwhile, the SIR model had stronger stability and avoided the problem of parameter identifiability than SEIAR (38). In addition, for the setting of susceptible individuals, we set all patients before 2010 to be susceptible. This is because HMFD was only incorporated into the infectious disease management system in China in 2008, and thus data on the recovered population are not available. The proportion of patients before that time is very small compared to the 10 million population in Shenzhen, so we believe that the effect is weak. The omission of this population may have an impact on the results of the age group component of the study, which needs to be discussed in further studies in future.

The results of the goodness-of-fit tests revealed that our model fitted the reported data well, which means that our modeling procedure has good validity and the model can be used to calculate the transmission rate of each pathogen.

In terms of the overall pattern, *R*_*eff*_ was >1 in Shenzhen city most of the time and in all administrative district, indicating that HFMD is still prevalent in the city and the disease burden remains serious, requiring attention and prevention and control concerns, whilst *R*_*eff*_ in Longgang district showed a gradual upward trend from 2010 to 2016. This may be due to the fact that Longgang, as a newly developed administrative region, has experienced rapid growth in population size and density in recent years, whereas the development of health care resources has failed to match the rate of population growth, leading to a rise in the transmissibility of HFMD. In contrast, the *R*_*eff*_ values in other districts experienced ups and downs between 1 and 11 with no significant trend.

When comparing the *R*_*eff*_ of the three age groups, it is clear that EV71 is most transmissible in the 0–2 years old group, while the *R*_*eff*_ of the 3–5 years old group is perennially lower than the other two groups. This may be due to the relatively high concentration of activities in the community among infants aged 0–2 years. Therefore, the proportion of infants and young children in the exposed population is also higher, leading to more disease transmission in this age group, and also to a greater *R*_*eff*_ of EV71 in the 3–5 years and the over 5 years group.

The 3–5 years age group should show a greater transmissibility due to increased socialization, while the smaller *R*_*eff*_ may be due to certain hygienic habits developed after school. Also, today's society shows concern for the disease and the parents and kindergarten teachers give more hygiene protection. The higher *R*_*eff*_ in the over 5 years old group compared to the previous group may be related to the older age group of EV71 in recent years, leading to an increased exposure rate. It can be seen that the transmission coefficient of HFMD for children in the 3–5 years group started to increase year by year in 2016. The greater contribution of transmission of the 3–5 years group to the transmission of the 0–3 years group may also be related to the comprehensive two-child policy launched in 2015.

After the launch of the HFMD vaccine in 2016, it is observed that the *R*_*eff*_ also declined in the city and in all districts except Yantian District. The results of the TSIR model developed by JT. Wu et al. similarly suggest that a mass EV-A71 vaccination program for infants and young children might significantly reduce the overall burden of HFMD (22). In our study, differences in the transmission capacity of different age groups for around this time point could be observed. However, the peak in the number of cases after the launch of the vaccine in the autumn of 2017 was significantly higher than in previous years, which may be due to the significant increase in the number of children after the introduction of the two-child policy, resulting in an increase in the number of HFMD cases, and may also account for the higher values of β_21_ in the second-stage fitting results in 2017.

In summary, we estimate that the disease burden of HFMD in Shenzhen city is severe with varying disease trends across districts. There is variability in transmission in different age groups, all of which are slightly weaker, respectively, relative to the overall picture. In addition, vaccination efforts have been effective, but prevention and control of HFMD caused by enterovirus such as Cox A16 and others need to be further strengthened.

## Data Availability Statement

The raw data supporting the conclusions of this article will be made available by the authors, without undue reservation.

## Author Contributions

PL, JR, TC, and YN made substantial contributions to conception and design. PL, QL, JR, TC, and YN collected the data and conceived the experiments. PL, QL, YW, FX, JR, ZL, and YN conducted the experiments and analyzed the results. JH, PL, JR, BD, LL, CL, WL, FX, TY, and YW involved in the visualization of the results. PL, JR, SY, CL, YN, and YW wrote the manuscript. QL, JR, PL, TC, and YN revised it critically for important intellectual content. All authors approved the final manuscript and agreed to be accountable for all aspects of the work.

## Funding

This study was partly supported by the Bill & Melinda Gates Foundation (INV-005834).

## Conflict of Interest

The authors declare that the research was conducted in the absence of any commercial or financial relationships that could be construed as a potential conflict of interest.

## Publisher's Note

All claims expressed in this article are solely those of the authors and do not necessarily represent those of their affiliated organizations, or those of the publisher, the editors and the reviewers. Any product that may be evaluated in this article, or claim that may be made by its manufacturer, is not guaranteed or endorsed by the publisher.
